# The regulation of M_1_ muscarinic acetylcholine receptor desensitization by synaptic activity in cultured hippocampal neurons^1^

**DOI:** 10.1111/j.1471-4159.2007.04931.x

**Published:** 2007-12

**Authors:** Jonathon M Willets, Carl P Nelson, Stefan R Nahorski, R A John Challiss

**Affiliations:** Department of Cell Physiology and Pharmacology, University of LeicesterLeicester, UK

**Keywords:** G protein-coupled receptor kinase, hippocampal neuron, muscarinic acetylcholine receptor, protein kinase C, receptor desensitization, synaptic activity

## Abstract

To better understand metabotropic/ionotropic integration in neurons we have examined the regulation of M_1_ muscarinic acetylcholine (mACh) receptor signalling in mature (> 14 days *in vitro*), synaptically-active hippocampal neurons in culture. Using a protocol where neurons are exposed to an EC_50_ concentration of the muscarinic agonist methacholine (MCh) prior to (R1), and following (R2) a desensitizing pulse of a high concentration of this agonist, we have found that the reduction in M_1_ mACh receptor responsiveness is decreased in quiescent (+tetrodotoxin) neurons and increased when synaptic activity is enhanced by blocking GABA_A_ receptors with picrotoxin. The picrotoxin-mediated effect on M_1_ mACh receptor responsiveness was completely prevented by α-amino-3-hydroxy-5-methyl-4-isoxazolepropionic acid receptor blockade. Inhibition of endogenous G protein-coupled receptor kinase 2 by transfection with the non-G_q/11_α-binding, catalytically-inactive ^D110A,K220R^G protein-coupled receptor kinase 2 mutant, decreased the extent of M_1_ mACh receptor desensitization under all conditions. Pharmacological inhibition of protein kinase C (PKC) activity, or chronic phorbol ester-induced PKC down-regulation had no effect on agonist-mediated receptor desensitization in quiescent or spontaneously synaptically active neurons, but significantly decreased the extent of receptor desensitization in picrotoxin-treated neurons. MCh stimulated the translocation of diacylglycerol- sensitive eGFP-PKCε, but not Ca^2+^/diacylglycerol-sensitive eGFP-PKCβII in both the absence, and presence of tetrodotoxin. Under these conditions, MCh-stimulated eGFP-myristoylated, alanine-rich C kinase substrate translocation was dependent on PKC activity, but not Ca^2+^/calmodulin. In contrast, picrotoxin-driven translocation of myristoylated, alanine-rich C kinase substrate was accompanied by translocation of PKCβII, but not PKCε, and was dependent on PKC and Ca^2+^/calmodulin. Taken together these data suggest that the level of synaptic activity may determine the different kinases recruited to regulate M_1_ mACh receptor desensitization in neurons.

Cholinergic innervation within the hippocampus originates mainly from the medial septal nuclei forming direct synaptic inputs to both principal neurons and interneurons throughout the dentate gyrus and CA3 and CA1 regions (Dutar *et al.* 1995; Rouse *et al.* 1999). There is also evidence that some cholinergic terminals do not associate with specific postsynaptic sites, indicative of additional diffuse modulator influences (Vizi and Kiss 1998). Effective transmission of cholinergic input is mediated by nicotinic and muscarinic acetylcholine (mACh) receptors, with the M_1_ and M_3_ mACh receptor subtypes being expressed on principal neurons (Levey *et al.* 1995). A major function of cholinergic transmission is to enhance the excitability of the hippocampal glutamate circuitry (Dutar *et al.* 1995) via a number of ionic conductances and second messenger systems (Cobb and Davies 2005) suggesting a role in long-term excitability and synaptic plasticity. Indeed, there is recent strong evidence for modulation of synaptic plasticity by the physiological activation of hippocampal M_1_ mACh receptors (Shinoe *et al.* 2005) and cholinergic neurotransmission has long been associated with cognitive processes, such as learning and memory (Blokland 1995; Fadda *et al.* 1996; Segal and Auerbach 1997).

M_1_ mACh receptors are the major subtype signalling through G_q/11_ to phospholipase C (PLC) and phosphoinositide hydrolysis in the hippocampus (Porter *et al.* 2002) and we have provided direct evidence for this signalling using fluorescence imaging approaches in single hippocampal neurons (Nash *et al.* 2004; Willets *et al.* 2004, 2005; Young *et al.* 2005). We have also already provided substantial evidence that M_1_ mACh receptor/PLC signalling in hippocampal neurons is regulated by G protein-coupled receptor kinases (GRKs). GRKs are widely expressed in the brain and we have identified GRKs 2, 3, 5 and 6 in our hippocampal cultures (Willets *et al.* 2004). However, there is still little direct evidence for their role in neuronal signalling (Willets *et al.* 2003; Gainetdinov *et al.* 2004). Endogenous GRK6 regulates M_1_ mACh receptors through a phosphorylation-dependent mechanism in hippocampal neurons, whilst endogenous GRK2 is able to use both phosphorylation-dependent and -independent (through the direct interaction of the regulator of G-protein signalling-like domain on the N-terminus of GRK2 and GTP-bound Gα_q/11_) mechanisms to regulate M_1_ mACh receptors in PLC signalling and desensitization (Willets *et al.* 2004, 2005). In the current study we have investigated mechanisms of regulation in mature hippocampal neurons that form synaptic interconnexions in culture (Bacci *et al.* 1999) and in which glutamate-mediated synaptic activity can be dramatically increased by suppressing inhibitory GABAergic inputs with picrotoxin, or suppressed with tetrodotoxin (TTx) (Bacci *et al.* 1999; Nash *et al.* 2004; Young *et al.* 2005). Indeed, previous studies have revealed that the level of synaptic activity in such cultures dramatically alters M_1_ mACh receptor inositol 1,4,5-trisphosphate generation and Ca^2+^ release from stores (Nash *et al.* 2004), and there is much evidence that mACh receptors can modulate oscillation frequency in hippocampal networks (see Cobb and Davies 2005). Our current data suggest that increased synaptic activity enhances hippocampal M_1_ mACh receptor desensitization through mechanisms involving endogenous GRK2 and Ca^2+^-sensitive protein kinase C (PKC) isoenzyme(s).

## Materials and methods

### Materials

Cell culture supplies and lipofection reagents were obtained from Invitrogen (Paisley, UK). Thermolysin, pronase, DNase I, poly-d-lysine, cytosine arabinoside and methacholine (MCh) were from Sigma-Aldrich (Poole, UK). Tocris (Bristol, UK) supplied picrotoxin, TTx, DNQX and D-AP5. Staurosporine and Ca^2+^/calmodulin (CaM) inhibitors W5 and W7 were obtained from Merck Biosciences/Calbiochem (Nottingham, UK).

### Cell culture and transfections

Hippocampal cultures were prepared as described previously (Nash *et al.* 2004; Willets *et al.* 2004). Briefly, isolated hippocampi from humanely killed 1-day-old Lister Hooded rats were dissociated with pronase E (0.5 mg/mL) and thermolysin (0.5 mg/mL) in a HEPES-buffered salt solution [HEPES-buffered salt solution (in mmol/L): NaCl, 130; HEPES, 10; KCl, 5.4; MgSO_4_, 1.0; glucose, 25; and CaCl_2_, 1.8, pH 7.2] for 30 min. Tissue fragments were further dissociated by trituration in HBSS containing DNase I (40 μg/mL). Following centrifugation and further trituration, cells were plated onto poly-d-lysine (50 μg/mL)-treated 25 mm glass coverslips. For the first 72 h cells were cultured in Neurobasal medium (Invitrogen), supplemented with B27, 10% fetal calf serum and penicillin (100 U/mL)/streptomycin (100 μg/mL). Cytosine arabinoside (5 μmol/L) was added after 24 h, and after 72 h cells were transferred to serum-free medium. Neurons were transfected after 11 days *in vitro*, using Lipofectamine 2000 reagent, following the manufacturer’s instructions. For experiments involving GRK constructs neurons were transfected with a 3 : 1 ratio of GRK or vector control to PH domain of PLCδ tagged to enhanced green fluorescent protein (eGFP-PH_PLCδ_). In all other cases 0.5 μg of cDNA was used per transfection. As reported previously, under these conditions mixed neuronal/glial cultures are generated, with the two populations being clearly distinguishable for imaging purposes (Young *et al.* 2005). Transfection efficiencies of the neuronal population were typically 2–4% (Willets *et al.* 2004).

### Measurement of inositol 1,4,5-trisphosphate in single cells and assessment of mACh receptor desensitization

Translocation of eGFP-PH_PLCδ_ was visualized using an Olympus FV500 scanning laser confocal IX70 inverted microscope. Cells were incubated at 37°C using a temperature controller and microincubator (PDMI-2 and TC202A; Burleigh, UK) and perfused at 5 mL/min with Krebs buffer (in mmol/L: NaCl 130, KCl 5.4, MgCl_2_ 1.0, HEPES 10, glucose 10, and CaCl_2_ 1.8, pH 7.4). Images were captured using an oil immersion ×60 objective. Activation of PLC, reflecting an increase in cytosolic inositol 1,4,5-trisphosphate (IP_3_) and/or a decrease in phosphatidylinositol 4,5-bisphosphate, was measured as the relative change in fluorescence detected in an area of interest as described previously (Nash *et al.* 2002, 2004; Willets *et al.* 2004). Changes in fluorescence are expressed as F/F_0_, where F is the fluorescence at a particular time, and F_0_ is the initial basal fluorescence. Drugs were applied via perfusion lines. Desensitization of the mACh receptor was assessed in hippocampal neurons transfected with eGFP-PH_PLCδ_ on day 11 *in vitro* and used experimentally between days 14 to 21 *in vitro*. Desensitization was assessed in single cells as described previously (Willets *et al.* 2004, 2005). Neurons were challenged with an approx. EC_50_ concentration of the mACh receptor agonist MCh (10 μmol/L) for 30 s (termed R1) followed by a 5 min washout to allow recovery. Following this a maximal concentration of MCh (100 μmol/L) was applied for 1 min to induce receptor desensitization. The washout period following desensitization was varied prior to re-challenge with the same approx. EC_50_ concentration of MCh (termed R2). Receptor desensitization was determined as the reduction in peak eGFP-PH_PLCδ_ translocation in R2 when compared to R1.

### Measurement of eGFP-myristoylated, alanine-rich C kinase substrate translocation

Neurons were transfected as described above with 0.5 μg of eGFP-tagged myristoylated, alanine-rich C kinase substrate (MARCKS). Imaging of eGFP-MARCKS translocation was undertaken as described for the eGFP-PH_PLCδ_ probe and was determined as the increase in cytosolic fluorescence following drug addition (Bartlett *et al.* 2005).

### Measurement of translocation of eGFP-PKC isoenzymes

Neurons were transfected with 0.5 μg of either eGFP-PKCβII or eGFP-PKCε. Translocation of eGFP-PKCs was assessed as described for eGFP-PH_PLCδ_. eGFP-PKC translocation was determined as a decrease in the cytosolic fluorescence as eGFP-PKC localized to the plasma membrane (Bartlett *et al.* 2005).

### Measurement of intracellular [Ca^2+^]

For dual measurement of PKC translocations and intracellular [Ca^2+^], neurons were transfected with eGFP-PKCε or eGFP-PKCβII and loaded with Fura-Red (3 μmol/L, 60 min) (Invitrogen, Paisley, UK) prior to the start of experiments. eGFP-PKCs and Fura-Red were excited via the 488 nm line of the argon laser. Fluorescence emissions from eGFP-PKCs and Fura-Red were collected at 505–560 nm and > 600 nm, respectively. Changes in eGFP-PKC fluorescence were calculated as described above, whilst increases in intracellular Ca^2+^ are reported as a decrease in Fura-Red fluorescence.

### Data analysis

Data were analysed using Prism 4 (GraphPad Software Inc., San Diego, CA, USA) and statistical analysis performed using one- or two-way analysis of variance (Excel 5.0; Microsoft, Redmond, WA, USA) where appropriate, followed by Student’s *t-*test or an alternative *post hoc* test stated in the text. Significance was accepted when *p*< 0.05.

## Results

### Effects of synaptic activity on M_1_ mACh receptor desensitization

**Fig. 1:**
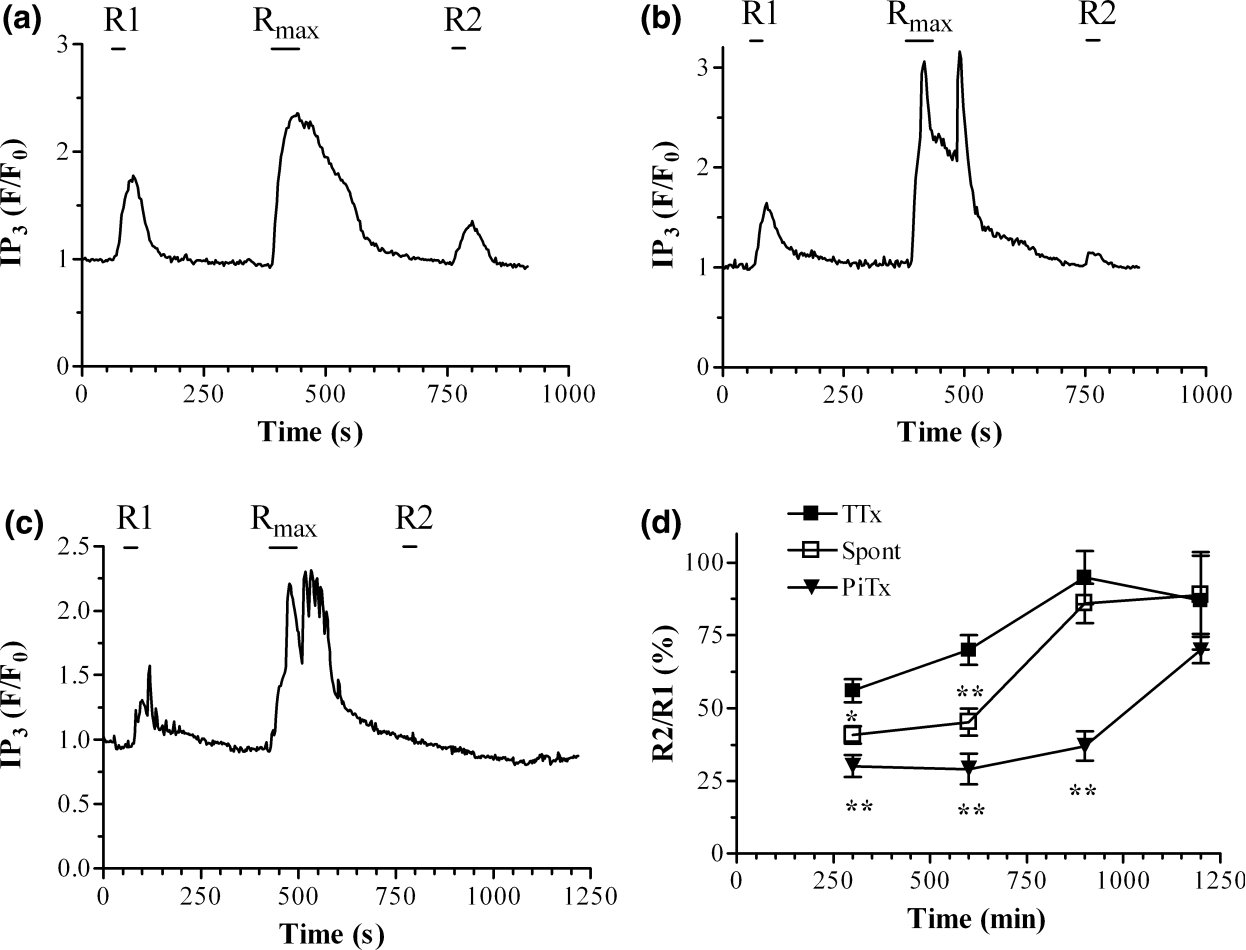
Effects of synaptic activity on M_1_ muscarinic acetylcholine (mACh) receptor responsiveness and re-sensitization assessed through inositol 1,4,5-trisphosphate (IP_3_) imaging of hippocampal neurons. The desensitization protocol (R1/R_max_/R2) was performed as described in the Experimental Procedures section. Representative traces showing M_1_ mACh receptor desensitization in the presence of (a) tetrodotoxin (TTx) (500 nmol/L); (b) no pre-addition (spontaneous synaptic activity; Spont in panel d); and (c) synaptic activity induced by picrotoxin (PiTx, 100 μmol/L). Picrotoxin was present 3 min prior to and throughout the experiment. Methacholine (R1, 10 μmol/L, 30 s; R_max_, 100 μmol/L, 60 s; R2 10 μmol/L, 30 s) was added as indicated by the bars. (d) Cumulative data for time-courses of M_1_ mACh receptor re-sensitization in the absence (Spont) or presence of TTx, or following PiTx addition. Data are expressed as means ± SEM for the percentage change in R2 relative to the R1 response, for 5–15 neurons taken from at least three separate hippocampal preparations. Significant differences in the R2/R1 ratio from the +TTx condition at a given time-point are indicated as *p< 0.05; **p< 0.01.

Our previous work has extensively characterized the desensitization of M_1_ mACh receptors in immature (< 10 days *in vitro*, hereafter DIV), non-synaptically active hippocampal neurons (Willets *et al.* 2004, 2005). Based on these findings we applied a similar protocol (see Materials and methods) to study receptor desensitization in mature (≥ 14 DIV) neurons. Comparison of the responses before (R1) and following (R2), the addition of a desensitizing pulse of MCh (100 μmol/L), showed a clear reduction in the R2 response 5 min after the desensitizing pulse under all conditions. In the presence of TTx (500 nmol/L; to block spontaneous synaptic activity), desensitization, indicated as the decrease in R2 response relative to the R1 response, was similar to our previous findings in immature neurons (Fig. 1a). However, in the absence of TTx the hippocampal cultures display spontaneous, glutamate-driven and α-amino-3-hydroxy-5-methyl-4-isoxazolepropionic acid (AMPA) receptor-mediated synaptic activity (Bacci *et al.* 1999; Nash *et al.* 2004; Young *et al.* 2005), which results in AMPA receptor-mediated depolarization and Ca^2+^ entry via NMDA receptors and voltage-operated Ca^2+^ channels. In agreement with our previous data (Nash *et al.* 2004) such activity promotes enhanced IP_3_ formation, seen as spikes above the level of IP_3_ produced by the presence of agonist (Fig. 1b). However, in the presence of spontaneous synaptic activity the difference between R2 and R1 responses was increased (Fig. 1b), suggesting that synaptic activity may increase the rate/extent of M_1_ mACh receptor desensitization. To test this hypothesis, we enhanced spontaneous synaptic activity by adding picrotoxin (100 μmol/L) to block GABA_A_ receptors and enhance the effect of glutamate-driven AMPA receptor-mediated Ca^2+^ excitability within the neuronal culture (Nash *et al.* 2004; Young *et al.* 2005). Picrotoxin (100 μmol/L) was added 3 min prior to the start of, and throughout the experiment. The presence of picrotoxin significantly suppressed the R2 response compared to R1, indicating that the level of synaptic activity within the neuronal culture directly effects receptor responsiveness (Fig. 1c).

### Effects of synaptic activity on M_1_ mACh receptor re-sensitization

We further examined the time period between desensitization and R2 application to assess the effects of synaptic activity on M_1_ mACh receptor re-sensitization. In the presence of TTx (500 nmol/L), R2 responses began to approach R1 responses following a 10 min recovery period (delay time between R_max_ and R2; see Fig. 1). No further re-sensitization was seen even following 30 min washout (data not shown). In the absence of TTx, R2 responses were slower to recover following the R_max_ stimulus, and full recovery did not occur until 15 min post-desensitization (Fig. 1d). When synaptic activity was enhanced with picrotoxin, receptor-stimulated PLC responses were significantly depressed for up to 15 min post-R_max_ (Fig. 1d). These data suggest that the degree of synaptic activity also regulates the duration of agonist-mediated desensitization, and a delay in M_1_ mACh receptor re-sensitization is particularly evident when inhibitory GABAergic activity is blocked by picrotoxin.

### Effects of ionotropic glutamate receptor blockade on picrotoxin-enhanced M_1_ mACh receptor desensitization

**Fig. 2:**
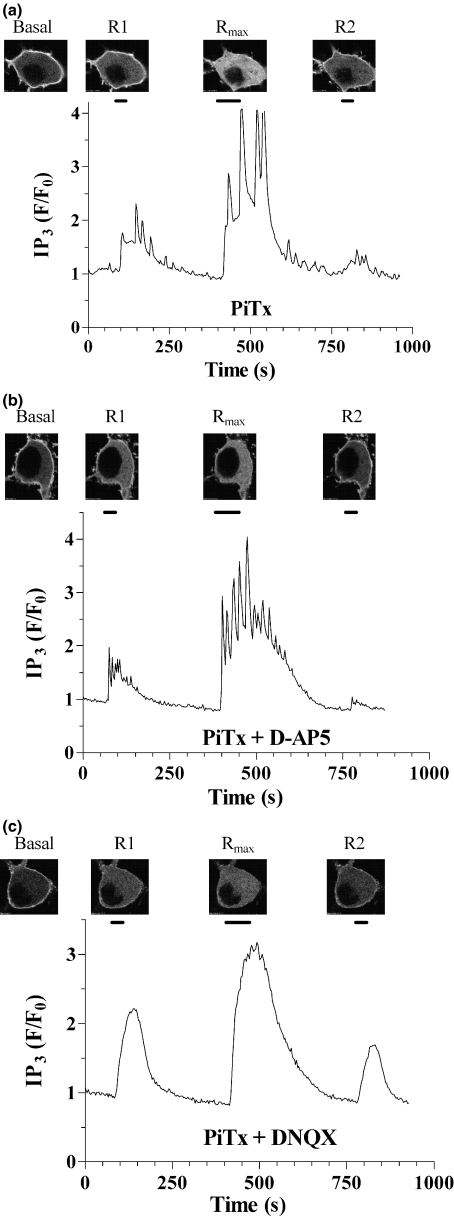
Effect of inhibition of α-amino-3-hydroxy-5-methyl-4-isoxazolepropionic acid and NMDA receptors on M_1_ muscarinic acetylcholine receptor desensitization. Representative images and traces showing M_1_ muscarinic acetylcholine receptor desensitization in the presence of (a) picrotoxin (PiTx, 100 μmol/L); (b) PiTx (100 μmol/L) + D-AP5 (50 μmol/L); (c) PiTx (100 μmol/L) + DNQX (10 μmol/L). Picrotoxin ± D-AP5/DNQX were present 3 min prior to R1 methacholine addition and throughout the experiment. methacholine (R1, 10 μmol/L, 30 s; R_max_, 100 μmol/L, 60 s; R2 10 μmol/L, 30 s) was added as indicated by the bars. Data are representative of 8–17 neurons taken from at least three separate hippocampal preparations (see Table 1).

In order to determine the relative roles of ionotropic glutamate receptor subtypes on enhanced glutamate-driven synaptic activity, we performed standard desensitization experiments in the presence or absence of pharmacological blockers of AMPA and NMDA receptors. Inhibition of NMDA receptors using D-AP5 (50 μmol/L) had no effect on the picrotoxin-enhanced M_1_ mACh receptor desensitization (Fig. 2b; Table 1). However, the picrotoxin-induced IP_3_ spikes normally seen in the presence of MCh (Fig. 2c) were absent following inclusion of the AMPA receptor antagonist DNQX (10 μmol/L). Furthermore, the presence of DNQX completed ablated the effects of picrotoxin treatment on M_1_ mACh receptor desensitization (Table 1), producing traces that were similar to those generated in the presence of TTx (Fig. 2c). These data suggest that the enhancement of M_1_ mACh receptor desensitization by picrotoxin is dependent on AMPA receptor activation.

**Table 1:** Inhibition of α-amino-3-hydroxy-5-methyl-4-isoxazolepropionic acid, but not NMDA receptors prevents picrotoxin-mediated enhancement of M_1_ muscarinic acetylcholine receptor desensitization

Treatment	R2/R1 (%)
TTx (500 nmol/L)	59.8 ± 4.0 (17)
TTx (500 nmol/L) + DNQX	53.4 ± 3.6 (8)
picrotoxin (100 μmol/L)	44.9 ± 3.6 (17)^a^
picrotoxin (100 μmol/L) + DNQX	59.2 ± 5.0 (12)^b^
picrotoxin (100 μmol/L) + D-AP5	38.8 ± 7.3 (9)

Data are expressed as means ± SEM for the percentage change in R2 relative to R1 responses for 8–17 neurons taken from at least three separate hippocampal preparations. In the presence of picrotoxin-induced activity, the R2/R1 ratio was reduced when compared to tetrodotoxin (TTx)-treated neurons (^a^*p < 0.05*; one-way anova, Dunnett’s *post-hoc* test). In the presence of DNQX (10 μmol/L), the effect of picrotoxin was significantly reversed (^b^*p*< 0.05; one-way anova, Dunnett’s *post-hoc* test), while D-AP5 (50 μmol/L) was without effect on the picrotoxin-mediated enhancement of M_1_ muscarinic acetylcholine receptor desensitization.

### Can increased synaptic activity alone induce receptor desensitization?

In order to assess whether synaptic activity was able to enhance M_1_ mACh receptor desensitization in the absence of MCh, we applied the following protocol. Neurons were stimulated with MCh (10 μmol/L) for 30 s, followed by a 5 min washout period. Then neurons were challenged with picrotoxin (100 μmol/L) or vehicle for 4 min. Following a further 5 min washout period neurons were again stimulated with MCh (10 μmol/L). Receptor desensitization was assessed as the decrease in R2 response when compared to R1. Under these conditions picrotoxin failed to produce any change in the R2/R1 ratio, indicating that a burst of synaptic activity alone is unable to induce M_1_ mACh receptor desensitization (data not shown).

### Involvement of GRKs in M_1_ mACh receptor desensitization

**Fig. 3:**
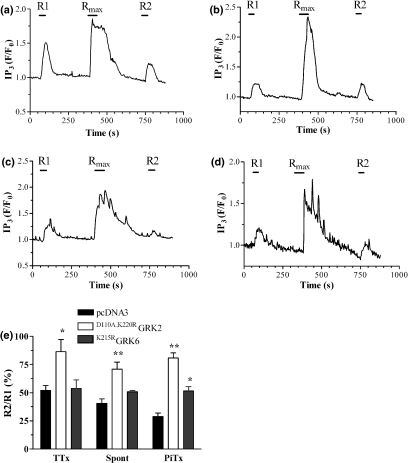
Effects of inhibiting endogenous G protein-coupled receptor kinase (GRK) 2 and GRK6 activities on M_1_ muscarinic acetylcholine (mACh) receptor responsiveness. Neurons were co-transfected with PH domain of PLCδ tagged to enhanced green fluorescent protein and empty vector (pcDNA3), ^D110A,K220R^GRK2, or ^K215R^GRK6. A standard R1/R_max_/R2 protocol was applied to assess M_1_ mACh receptor desensitization in either the absence (spontaneous activity; spont) or presence of tetrodotoxin (TTx, 500 nmol/L), or in the presence of picrotoxin (PiTx, 100 μmol/L). Representative traces showing M_1_ mACh receptor desensitization in the presence of (a) pcDNA3 and TTx; (b) ^D110A,K220R^GRK2 and TTx; (c) pcDNA3 and PiTx; and (d) ^D110A,K220R^GRK2 and PiTx. Methacholine addition is indicated by the horizontal bars. (e) cumulative data are presented as means ± SEM for the percentage change in R2 relative to the R1 response, for five neurons for each treatment taken from at least three separate hippocampal preparations. Significant differences in the R2/R1 ratio caused by expression of the dominant-negative ^D110A,K220R^GRK2 or ^K215R^GRK6 constructs are indicated as *p< 0.05; **p< 0.01.

To determine how inhibition of endogenous GRKs 2 and/or GRK6 alters M_1_ mACh receptor desensitization, neurons were co-transfected with catalytically-inactive (dominant-negative) versions of GRK2 (^D110A,K220R^GRK2) or ^K215R^GRK6 and eGFP-PH_PLCδ_ (Willets *et al.* 2004, 2005). The GRK2 construct also possesses a mutation (D110A) in its regulator of G-protein signalling-like domain, which prevents binding to GTP-bound Gα_q_, allowing the study of GRK-mediated/receptor interactions without direct inhibition of Gα_q_ activation of PLC (Willets *et al.* 2005). Co-transfection of eGFP-PH_PLCδ_ and pcDNA3 did not affect the pattern of M_1_ mACh receptor desensitization, which was again enhanced in the presence of picrotoxin (Fig. 3). In ^D110A,K220R^GRK2-transfected neurons the R2/R1 ratio difference was dramatically reduced indicating that inhibition of endogenous GRK2 attenuates M_1_ mACh receptor desensitization under all conditions, including those where the extent of receptor desensitization was increased by picrotoxin treatment (see Fig. 3e). In contrast, expression of ^K215R^GRK6 did not affect M_1_ mACh receptor desensitization in TTx-treated, or spontaneously synaptically-active neurons (Fig. 3e). However, inhibition of endogenous GRK6 appeared to partially prevent picrotoxin-enhanced M_1_ mACh receptor desensitization (Fig. 3e).

### Involvement of PKC in M_1_ mACh receptor desensitization

**Fig. 4:**
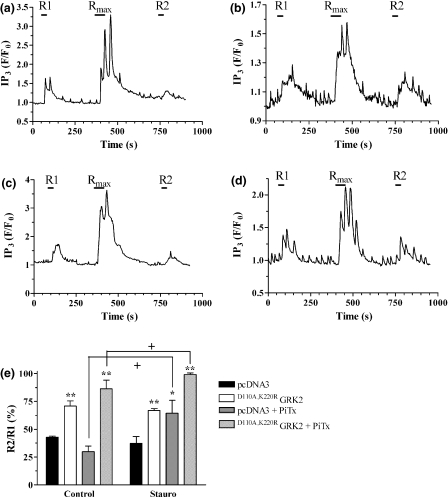
Effects of manipulating protein kinase C activity in hippocampal neurons on M_1_ muscarinic acetylcholine receptor responsiveness. Neurons were co-transfected with PH domain of PLCδ tagged to enhanced green fluorescent protein and empty vector (pcDNA3) or ^D110A,K220R^G protein-coupled receptor kinase (GRK) 2 and changes in receptor responsiveness determined using the standard R1/R_max_/R2 protocol (see Experimental Procedures). Neurons were pre-incubated with either vehicle (Control) or staurosporine (1 μmol/L; Stauro) for 15 min prior to, and throughout the experiment. Representative traces are shown for neurons treated with picrotoxin (PiTx, 100 μmol/L) in the presence of (a) pcDNA3 and vehicle; (b) ^D110A,K220R^GRK2 and vehicle; (c) pcDNA3 + staurosporine (1 μmol/L); (d) ^D110A,K220R^GRK2 and staurosporine (1 μmol/L). (e) cumulative data are shown for either spontaneously active or picrotoxin-treated neurons and are expressed as means ± SEM for the percentage change in R2 relative to the R1 response, for ≥ 5 neurons per treatment taken from at least three separate hippocampal preparations. Significant differences in the R2/R1 ratio caused by either ^D110A,K220R^GRK2 expression are indicated as *p< 0.05; **p< 0.01, while a significant effect of staurosporine pre-treatment is shown as ^+^*p*< 0.05.

To assess whether PKC plays a role in M_1_ mACh receptor desensitization we pre-incubated neurons with vehicle (0.01% dimethylsulphoxide, hereafter DMSO) or the PKC inhibitor staurosporine (1 μmol/L) for 15 min. Next neurons were subjected to the standard desensitization R1/R_max_/R2 protocol, in the presence of vehicle or staurosporine throughout the experiment. In spontaneously synaptically-active, pcDNA3-transfected cultures PKC inhibition had no effect on the R2/R1 ratio, implying that PKC does not play a role in agonist-stimulated M_1_ mACh receptor desensitization (Fig. 4a and e). In addition, staurosporine did not alter the R2/R1 ratio in the presence of ^D110A,K220R^GRK2 in spontaneously synaptically-active neurons. In contrast, staurosporine pre-treatment had a significant effect on the R2/R1 ratio when synaptic activity was enhanced in the presence of picrotoxin (Fig. 4b and e). Furthermore, staurosporine (1 μmol/L) pre-treatment was also able to enhance the effect of ^D110A,K220R^GRK2 expression, resulting in an almost complete blockade of M_1_ mACh receptor desensitization in picrotoxin-treated neurons (see Fig. 4e). In agreement with the staurosporine data, down-regulation of PKC isoenzymes by a 24 h pre-treatment with phorbol 12,13-dibutyrate (1 μmol/L) significantly attenuated the decrease in R2/R1 ratio caused by agonist challenge in picrotoxin-treated neurons, but was without apparent effect in TTx-treated, or spontaneously-active neurons (data not shown). While neither staurosporine nor chronic phorbol ester treatments act specifically on PKCs in neurons, the fact that these two interventions produce essentially identical data strongly implicate PKCs as the kinases that may additionally regulate M_1_ mACh receptor responsiveness under conditions of enhanced synaptic activity. Thus, these data are strongly suggestive that under conditions of picrotoxin-enhanced synaptic activity, GRK2 and PKCs act together to enhance M_1_ mACh receptor desensitization.

### *In situ* assessment of PKC activity using eGFP-MARCKS

**Fig. 5:**
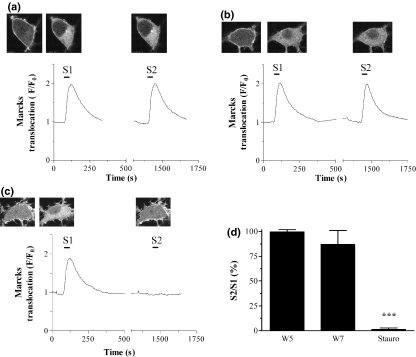
Effects of Ca^2+^/calmodulin antagonism or protein kinase C inhibition on M_1_ muscarinic acetylcholine receptor-driven myristoylated, alanine-rich C kinase substrate (MARCKS) translocation in hippocampal neurons. Neurons were transfected with enhanced green fluorescent protein (eGFP)-MARCKS. Agonist addition [methacholine (MCh), 3 μmol/L] for 30 s induced a robust translocation of eGFP-MARCKS from the plasma membrane to the cytoplasm, which completely reversed after agonist removal. Following the initial MCh stimulation (S1), neurons were treated with W5 (25 μmol/L; panel a), W7 (25 μmol/L; panel b), or staurosporine (1 μmol/L; panel c) for 15 min, prior to a second application of MCh (3 μmol/L, for 30 s; S2). Panels (a)–(c) present representative traces with accompanying images. (d) cumulative data showing the effect of W5, W7 and staurosporine (Stauro) on the percentage change in S2 relative to the S1 for MCh-stimulated eGFP-MARCKS translocation. Data are expressed as means ± SEM for at least four neurons per treatment taken from three separate hippocampal preparations. The statistically significant effect of staurosporine treatment on the S2/S1 ratio is indicated as ***p< 0.001.

To assess whether synaptic activity is able to promote PKC activation we first transfected neurons with an eGFP-MARCKS construct. This protein is membrane-associated and translocates to the cytoplasm when phosphorylated by PKCs or bound to CaM (Graff *et al.* 1989; Arbuzova *et al.* 2002). Stimulation of M_1_ mACh receptors with MCh (3 μmol/L) produced a rapid and transient translocation of MARCKS to the cytoplasm, which quickly returned to the plasma membrane on agonist removal. To determine whether PKC or Ca^2+^/CaM were responsible for M_1_ mACh receptor-mediated MARCKS translocation the following protocol was used. Neurons were stimulated with MCh (3 μmol/L) for 30 s (S1) and then washed to allow recovery of MARCKS to baseline levels. Neurons were then treated with staurosporine (1 μmol/L, to inhibit PKC; Fig. 5c), vehicle (0.01% DMSO), or the CaM inhibitor W7 (25 μmol/L; Fig 5b), for 15 min. Some neurons were treated with W5 (25 μmol/L), the inactive analogue of W7 (Fig. 5a). Neurons were then subjected to a second MCh (3 μmol/L, 30 s) challenge (S2). Comparison of S1 and S2 responses indicated that MCh-stimulated eGFP-MARCKS translocation was almost completely inhibited following staurosporine treatment (Fig. 5c and d), while W7 and W5 treatments were without effect (Fig. 5d). These data suggest that agonist-driven eGFP-MARCKS translocation is mediated through PKC activation in hippocampal neurons.

**Fig. 6:**
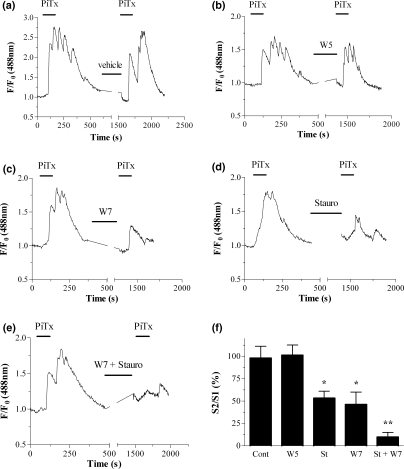
Effects of Ca^2+^/calmodulin antagonism and/or protein kinase C inhibition on picrotoxin (PiTx)-induced myristoylated, alanine-rich C kinase substrate translocation. Neurons were transfected with enhanced green fluorescent protein-myristoylated, alanine-rich C kinase substrate. Picrotoxin (100 μmol/L) was initially added to neurons for 2 min (S1); following washout neurons were incubated with vehicle (a), W5 (25 μmol/L; b), W7 (25 μmol/L; c), staurosporine (1 μmol/L; d) or W7 and staurosporine together (e), for 15 min, followed immediately by a second challenge (S2) with picrotoxin (100 μmol/L) for 2 min. Panels (a)–(e) present representative traces. (f) cumulative data are expressed as means ± SEM for the percentage change in S2 relative to the S1 response, for 4–6 neurons for each condition taken from at least three separate hippocampal preparations. Statistically significant inhibitor effects on the S2/S1 ratio compared to vehicle control are indicated as *p< 0.05; **p< 0.01.

To determine whether synaptic activity alone could promote eGFP-MARCKS translocation, neurons were incubated with picrotoxin in the absence of agonist for 2 min. Picrotoxin (100 μmol/L) stimulated a rapid increase in eGFP-MARCKS translocation to the cytoplasm, which returned to baseline following picrotoxin removal (Fig. 6). We again applied the S1/S2 protocol for picrotoxin with 15 min treatments with vehicle (0.01% DMSO; Fig. 6a), W5 (25 μmol/L, Fig. 6b), W7 (25 μmol/L, Fig. 6c), or staurosporine (1 μmol/L, Fig. 6d), between applications of picrotoxin. Under these conditions, staurosporine treatment caused an approx. 50% decrease in the S2/S1 ratio; W7 treatment produced an equivalent decrease to that caused by staurosporine, while W5 was without effect. Addition of staurosporine and W7 together almost completely inhibited picrotoxin-stimulated eGFP-MARCKS translocation (Fig. 6e and f). These data indicate that the picrotoxin-mediated eGFP-MARCKS translocation occurs through both PKC- and Ca^2+^/CaM-dependent mechanisms.

### Effects of picrotoxin and M_1_ mACh receptor activation on the translocation of PKCβII and PKCε

**Fig. 7:**
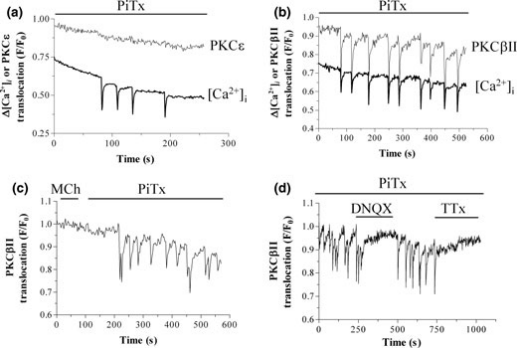
Translocation of conventional and novel protein kinase C (PKCs) following picrotoxin (PiTx) treatment of hippocampal neuron cultures. eGFP-PKCβII or eGFP-PKCε transfected neurons were challenged with PiTx (100 μmol/L) and PKC translocation to the plasma membrane determined as the decrease in cytosolic fluorescence. In panels (a) and (b), neurons were also loaded with the Ca^2+^-sensitive dye Fura-Red for 60 min prior to addition of PiTx. Note that downward deflections in the Ca^2+^ trace indicate increases in [Ca^2+^]_i_. (a) representative trace showing that picrotoxin addition alone did not stimulate translocation of eGFP-PKCε. (b) representative trace showing that, in contrast, PiTx addition alone caused transient increases in intracellular [Ca^2+^] that are mirrored by rapid and transient translocations of eGFP-PKCβII. (c) representative trace showing the effects of sequential methacholine (MCh, 100 μmol/L) and PiTx (100 μmol/L) additions on the translocation of eGFP-PKCβII. (d) representative trace showing the effects of the α-amino-3-hydroxy-5-methyl-4-isoxazolepropionic acid receptor blocker 6,7-dinitroquinoxaline-2,3-dione (DNQX, 10 μmol/L) and tetrodotoxin (TTx, 500 nmol/L) on PiTx-induced eGFP-PKCβII translocations. All representative traces are shown for experiments repeated on 4–10 coverslips from at least three separate hippocampal preparations.

The translocation of eGFP-MARCKS indicates that PKC activity can be stimulated in hippocampal neurons following stimulation of the M_1_ mACh receptor and also following picrotoxin-mediated enhancement of synaptic activity. To gain a better understanding of PKC isoenzymic recruitment patterns in neurons subject to metabotropic and/or ionotropic stimulation, we transfected neurons with eGFP-tagged constructs of either the Ca^2+^/diacylglycerol (DAG)-activated PKCβII, or the DAG-activated PKCε. Neurons, recombinantly expressing either eGFP-PKCβII or -ε isoenzymes and loaded with Fura-Red simultaneously to report changes in intracellular Ca^2+^ concentrations, were subject to picrotoxin and/or M_1_ mACh receptor agonist additions. Under these conditions it could be shown that picrotoxin treatment caused rapid and transient increases in intracellular Ca^2+^ (Fig. 7a and b). Perhaps unsurprisingly, picrotoxin addition did not cause the recruitment of the DAG-sensitive PKCε to the plasma membrane (Fig. 7a), however, it did produce rapid, transient recruitments of eGFP-PKCβII (Fig. 7b). Indeed, the translocation of eGFP-PKCβII precisely mirrored the picrotoxin-stimulated changes in intracellular Ca^2+^ (Fig. 7b). MCh (10 μmol/L) stimulation consistently failed to cause eGFP-PKCβII translocation, while addition of picrotoxin (100 μmol/L) induced rapid, transient translocations of eGFP-PKCβII (Fig. 7c). Further investigation showed that picrotoxin-induced eGFP-PKCβII translocations were inhibited completely following the addition of the AMPA receptor antagonist DNQX (10 μmol/L), and following blockade of synaptic activity by TTx (500 nmol/L) (Fig. 7d). These data suggest that eGFP-PKCβII translocations are driven by Ca^2+^ entry following AMPA receptor activation and voltage-operated Ca^2+^ channel opening.

**Fig. 8:**
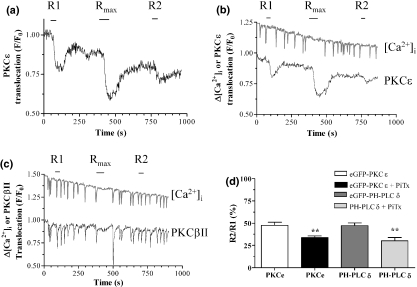
Effects of synaptic activity on M_1_ muscarinic acetylcholine receptor responsiveness assessed at the level of protein kinase C (PKC) isoenzyme recruitment to the plasma membrane in hippocampal neurons. Neurons were transfected with eGFP-PKCε or eGFP-PKCβII and subjected to the standard R1/R_max_/R2 protocol in the absence (a), or presence (b and c) of picrotoxin (PiTx, 100 μmol/L) for 3 min prior to, and for the duration of the experiment. Methacholine (R1, 10 μmol/L, 30 s; R_max_, 100 μmol/L, 60 s; R2 10 μmol/L, 30 s) was added as indicated by the bars. (a) representative trace showing the eGFP-PKCε translocations evoked during the methacholine stimulation protocol. (b) representative traces showing the effects of PiTx (100 μmol/L) on plasma membrane recruitment of eGFP-PKCε and concurrent changes in intracellular [Ca^2+^], during the receptor desensitization protocol. Note that downward deflections in the Ca^2+^ trace reflect increases in [Ca^2+^]_i_. (c) representative traces showing the coincident translocation in eGFP-PKCβII to the plasma membrane with changes in intracellular [Ca^2+^]. All representative traces are shown for experiments repeated on 5–8 coverslips from at least three separate hippocampal preparations. (d) cumulative data showing the effects of spontaneous activity and picrotoxin (100 μmol/L)-stimulated synaptic activity on M_1_ muscarinic acetylcholine receptor desensitization measured using the standard R1/R_max_/R2 protocol. Data are presented as means ± SEM for the % change in R2 relative to the R1 response, for 6–18 neurons for each treatment taken from at least three separate hippocampal preparations. Statistically significant effects of PiTx addition on the R2/R1 ratio are indicated as **p< 0.01 (Student’s *t*-test).

To determine the effects of the M_1_ mACh receptor desensitization protocol on plasma membrane PKC recruitment, we measured the translocation of eGFP-PKCε and eGFP-PKCβII during application of the R1/R_max_/R2 protocol (Fig. 8). This protocol produced robust, reversible MCh-stimulated membrane recruitments of eGFP-PKCε, which mirror data obtained with the eGFP-PH_PLCδ_ biosensor, suggesting that translocation of this probe is driven by changes in the concentration of DAG in the plasma membrane (Fig. 8a). Indeed, the decrease in R2/R1 ratio brought about by sequential MCh challenge was quantitatively similar using the eGFP-PKCε and eGFP-PH_PLCδ_ probes (Fig. 8d). In neurons expressing the eGFP-PKCε construct application of picrotoxin during the desensitization protocol caused a further suppression of R2 relative to R1 responses to MCh, similar in extent to that previously observed in neurons expressing eGFP-PH_PLCδ_ (Fig. 8b). These data indicate that eGFP-PKCε translocates in response to changes in DAG production and that these biosensors quantitatively report the same receptor desensitization phenomenon. To further investigate this we attempted to use the well-characterized DAG sensor tandem repeat of C1 domain of PKCγ tagged to enhanced green fluorescent protein (Oancea *et al.* 1998); however this construct was poorly expressed and may be toxic to our hippocampal neuronal cultures. Only very modest MCh-stimulated eGFP-PKCβII translocations were observed in neuronal cultures and these usually only occurred in response to challenge with high concentrations of MCh (≥ 100 μmol/L; data not shown).

For experiments assessing the effects of picrotoxin-evoked synaptic activity on PKC translocations, neurons were loaded with Fura-Red to allow co-determination of changes in intracellular [Ca^2+^]. Picrotoxin (100 μmol/L) addition produced rapid, transient rises in intracellular [Ca^2+^] (Fig. 8b and c). As reported above, the change in eGFP-PKCε translocation before and after a desensitizing MCh challenge can provide an readout of receptor desensitization, however, picrotoxin (100 μmol/L) addition *per se* did not promote eGFP-PKCε translocation, nor did it appear to affect agonist-mediated M_1_ mACh receptor regulation (Fig. 8b). In contrast, while agonist addition caused minimal translocation of eGFP-PKCβII, picrotoxin treatment (100 μmol/L) induced rapid, transient eGFP-PKCβII translocations to the plasma membrane, which mirrored the changes in intracellular [Ca^2+^] (Fig. 8c).

## Discussion

Recent studies from this laboratory have provided strong evidence that endogenous GRK2 and GRK6 can regulate the responsiveness of M_1_ mACh receptor signalling in cultured rat hippocampal neurons (Willets *et al.* 2004, 2005). Our approach has been to image the activation of PLC by this receptor using the eGFP-PH_PLCδ_ biosensor and co-transfection of wild-type, catalytically-inactive or antisense constructs to various kinases. In particular, we have established that GRK2 can suppress neuronal M_1_ mACh receptor signalling by both phosphorylation-dependent and -independent mechanisms with the latter probably involving the direct binding of the RH domain of GRK2 to Gα_q/11_ (Willets *et al.* 2005). Our previous studies were performed on immature neurons (≤ 8 DIV) before the development of spontaneous synaptic activity within the culture. Beyond ∼10 DIV these cultures form synaptic connections, which, via glutamatergic transmission, result in spontaneous, often synchronous bursts of action potentials that generate oscillatory changes in intracellular Ca^2+^ (Ogura *et al.* 1987; Bacci *et al.* 1999; Hardingham *et al.* 2001; Liu *et al.* 2003; Nash *et al.* 2004) which resembles, at least to some degree, the complex neuronal network activity observed in intact hippocampal preparations (LeBeau *et al.* 2005). We have also shown that activation of M_1_ mACh receptors can increase synaptic excitability in hippocampal neurons, probably via an agonist-mediated depletion of phosphatidylinositol 4,5-bisphosphate and activation of I_h_ (Young *et al.* 2005). Furthermore, enhanced synaptic activity in hippocampal neurons, initiated by suppression of inhibitory GABA_A_ receptors with picrotoxin, dramatically enhances M_1_ mACh receptor-stimulated IP_3_ production and Ca^2+^ store release (Nash *et al.* 2004). Moreover, very recent studies have revealed that conditions that mimic the *in vivo* environment of continuous synaptic bombardment of neocortical neurons show dramatically reduced mACh receptor agonist effects on membrane conductances (Desai and Walcott 2006).

In view of these data, we have examined M_1_ mACh receptor responsiveness in mature cultures of hippocampal neurons in which the level of synaptic activity has either been suppressed by TTx or enhanced by picrotoxin. Our data reveal clear evidence that agonist-mediated desensitization of M_1_ mACh receptor (with respect to the stimulation of PLC activity) is both enhanced and prolonged under conditions of increased synaptic activity. Furthermore, a combined action of GRK2 and Ca^2+^-dependent PKC(s) is indicated in the enhanced desensitization of M_1_ mACh receptor signalling observed in such synaptically active hippocampal neuronal cultures. Our current data also emphasize that the role of GRK2 in this regulation is dependent on its kinase activity. There is now much evidence that GRK2 can suppress a receptor-mediated Gα_q/11_ activation in a phosphorylation-independent manner through a specific interaction of GTP-loaded Gα_q/11_ with the RH domain located at the N-terminal of GRK2 (Carman *et al.* 1999; Sterne-Marr *et al.* 2003; Tesmer *et al.* 2005; Willets *et al.* 2005). In hippocampal neurons over-expression of GRK2, or its kinase-dead mutant form, completely suppresses M_1_ mACh receptor-stimulated PLC signalling (Willets *et al.* 2004). However, data obtained by expressing a dominant-negative GRK2 construct that also possesses a mutation (D110A) in the RH domain (preventing binding to GTP-loaded Gα_q/11_), suggest that the phosphorylation-dependent activity of endogenous GRK2 is required for M_1_ mACh receptor desensitization in synaptically-active hippocampal neurons (Willets *et al.* 2005).

The enhanced and prolonged desensitization of M_1_ mACh receptor signalling observed in picrotoxin- and agonist-treated cultures appears to depend on both GRK2 and PKC activities, as expression of the dominant-negative ^D110A,K220R^GRK2 construct only partially ameliorated agonist-induced receptor desensitization and further amelioration was observed in the presence of either a PKC inhibitor, or following phorbol ester-induced down-regulation of PKC activities. Moreover, our studies, using the translocation of eGFP-MARCKS have provided evidence for the activation of PKC in hippocampal neurons following M_1_ mACh receptor stimulation under conditions of increased synaptic activity. MARCKS is normally associated with the plasma membrane and translocates to the cytoplasm on phosphorylation by PKC and/or CaM binding (Graff *et al.* 1989; Arbuzova *et al.* 2002). Here we provide evidence that the translocation of eGFP-MARCKS stimulated by MCh is predominantly dependent on PKC activity, supporting our previous studies with M_3_ mACh receptors both in Chinese hamster ovary cells (Bartlett *et al.* 2005) and in cerebellar granule cells (Young *et al.* 2004). Increased synaptic activity induced by picrotoxin in the hippocampal neurons also resulted in the translocation of MARCKS and inhibitor studies implicate both a PKC and CaM regulatory component under these conditions. Indeed, MARCKS translocation in response to MCh was paralleled by a reciprocal movement of eGFP-PKCε, but not PKCβII, to the plasma membrane. In hippocampal neurons the M_1_ mACh receptor stimulates robust IP_3_/diacyglycerol accumulation, but only limited and transient intracellular Ca^2+^ signals. Increased synaptic activity induced by picrotoxin in the hippocampal neurons, however also resulted in the translocation of MARCKS, which was now accompanied by both Ca^2+^-dependent PKCβII and Ca^2+^-independent PKCε translocations. These results together with inhibitor studies strongly implicate PKC- and CaM-dependent components to MARCKS translocation under these conditions. Similar conclusions were reached in our previous studies on NMDA receptor-stimulated cerebellar granule cells (Young *et al.* 2004). Therefore, under conditions of picrotoxin-enhanced synaptic activity in hippocampal neurons, synchronous bursts of action potentials result in robust oscillatory changes in intracellular Ca^2+^ (Nash *et al.* 2004; Young *et al.* 2005) with consequent activation of CaM-dependent protein kinase and PKCs, as revealed by eGFP-MARCKS translocation. How this complex cross-talk results in an enhanced and prolonged M_1_ mACh receptor desensitization in synaptically-active neurons is not yet clear. Indeed, it should be emphasized that in immature, quiescent neurons neither Ca^2+^ nor PKC appear to be involved in agonist-evoked M_1_ mACh receptor desensitization (Willets *et al.* 2005). Whether independent/permissive phosphorylations of the M_1_ receptor by PKCs and GRK2 occur, or whether a synergistic modulation of GRK activity by PKC results in such regulation remains to be established. However, in relation to the latter, PKC has previously been shown to phosphorylate GRK2 resulting in enhanced desensitization of β_2_-adrenergic receptors (Chuang *et al.* 1995), and more recently phosphorylation of GRK2 by PKC was shown to suppress its inhibition by CaM (Krasel *et al.* 2001).

Whatever the mechanism(s), the present studies provide a novel insight into the regulation of endogenous G protein-coupled receptor activity in synaptically-active hippocampal neurons. As such it provides the first evidence that ionotropic glutamate receptor-mediated synaptic activity can dramatically alter M_1_ mACh receptor responsiveness by mechanisms involving both GRK2 and PKC(s) in primary neurons. What might be the consequences of greater and/or more sustained M_1_ mACh receptor desensitization? There is substantial evidence that GRK-mediated phosphorylation of mACh receptors brings about the recruitment of arrestins that enhance receptor internalization and lead to the redistribution of receptors to organellar compartments within the cytoplasm in primary neurons (Volpicelli and Levey 2004; Bernard *et al.* 2006). In addition, it is now well established that phosphorylated-receptor recruited arrestins can act as scaffolds for various signalling proteins, including components of the extracellular signal-regulated kinase/c-Jun N-terminal kinase cascades (Daaka *et al.* 1998; Lefkowitz and Shenoy 2005). It has also been shown previously that M_1_ mACh receptor stimulation can activate extracellular signal-regulated kinase in hippocampal neurons (Berkeley *et al.* 2001; Berkeley and Levey 2003), although it is not yet known whether this is GRK2/arrestin-dependent. Therefore, it is tempting to speculate that ionotropic modulation of the magnitude and/or longevity of receptor desensitization might not only lead to changes in plasmalemmal M_1_ mACh receptor expression, but also potentially lead to altered neuronal signal transduction through arrestin-dependent orchestration of key signalling pathways.

Our study has revealed novel information on the regulation of M_1_ mACh receptors by both agonist and synaptically activated excitability changes. Whether this represents an important mechanism of acute regulation of G protein-coupled receptor activity in neurons, or whether this increases the potential of the receptor to integrate with glutamate-mediated synaptic activity and, for example, signalling to the nucleus to influence dendritic function and plasticity, requires further investigation. However, in this regard Power and Sah (2002) have showed that following physiological activation of mACh receptors in hippocampal CA1 neurons, Ca^2+^ waves invade the nucleus by a mechanism dependent on IP_3_-sensitve stores. We have also shown that increased synaptic activity in hippocampal neurons results in a Ca^2+^-dependent enhancement of M_1_ mACh receptor-mediated PLC activity and increased Ca^2+^ store release (Nash *et al.* 2004). Finally, another recent study (Buttery *et al.* 2006) has shown that stimulation of hippocampal M_1_ mACh receptors (and mGlu receptors) recruits α1-chimaerin, a Rac-GTPase-activating protein, to the plasma membrane and its expression is highly dependent on the excitability of the neuronal culture. α1-chimaerin is known to modulate dendritic spine formation/removal in neurons (Van de Ven *et al.* 2005). Overall, this could provide a complex bi-directional cross-talk between ionotropic and metabotropic signalling in neurons that may underlie the regulation of longer-term modifications, such as synaptic plasticity.

## Abbreviations used

AMPA: α-amino-3-hydroxy-5-methyl-4-isoxazolepropionic acid
CaM: Ca^2+^/calmodulin
DAG: diacylglycerol
DIV: days *in vitro*
DMSO: dimethylsulphoxide
eGFP-PH_PLCδ_: PH domain of PLCδ tagged to enhanced green fluorescent protein
GRK: G protein-coupled receptor kinase
IP_3_: inositol 1,4,5-trisphosphate
mACh: muscarinic acetylcholine
MARCKS: myristoylated, alanine-rich C kinase substrate
MCh: methacholine
PKC: protein kinase C
PLC: phospholipase C
TTx: tetrodotoxin
